# The Role of Mitochondrial DNA Damage and Repair in the Resistance of BCR/ABL-Expressing Cells to Tyrosine Kinase Inhibitors

**DOI:** 10.3390/ijms140816348

**Published:** 2013-08-07

**Authors:** Sylwester Glowacki, Ewelina Synowiec, Janusz Blasiak

**Affiliations:** Department of Molecular Genetics, Faculty of Biology and Environmental Protection, University of Lodz, Pomorska 141/143, Lodz 90-236, Poland; E-Mails: sglowa@biol.uni.lodz.pl (S.G.); ewelinas@biol.uni.lodz.pl (E.S.)

**Keywords:** chronic myeloid leukemia, tyrosine kinase inhibitors, imatinib, apoptosis, mitochondrial DNA, DNA damage

## Abstract

Chronic myeloid leukemia (CML) is a hematological malignancy that arises from the transformation of stem hematopoietic cells by the fusion oncogene *BCR/ABL* and subsequent clonal expansion of BCR/ABL-positive progenitor leukemic cells. The BCR/ABL protein displays a constitutively increased tyrosine kinase activity that alters many regulatory pathways, leading to uncontrolled growth, impaired differentiation and increased resistance to apoptosis featured by leukemic cells. Current CML therapy is based on tyrosine kinase inhibitors (TKIs), primarily imatinib, which induce apoptosis in leukemic cells. However, some patients show primary resistance to TKIs while others develop it in the course of therapy. In both cases, resistance may be underlined by perturbations in apoptotic signaling in leukemic cells. As mitochondria may play an important role in such signaling, alteration in mitochondrial metabolism may change resistance to pro-apoptotic action of TKIs in BCR/ABL-positive cells. Because BCR/ABL may induce reactive oxygen species and unfaithful DNA repair, it may affect the stability of mitochondrial DNA, influencing mitochondrial apoptotic signaling and in this way change the sensitivity of CML cells to TKIs. Moreover, cancer cells, including BCR/ABL-positive cells, show an increased level of glucose metabolism, resulting from the shift from oxidative phosphorylation to glycolysis to supply ATP for extensive proliferation. Enhanced level of glycolysis may be associated with TKI resistance and requires change in the expression of several genes regulated mostly by hypoxia-inducible factor-1α, HIF-1α. Such regulation may be associated with the impaired mitochondrial respiratory system in CML cells. In summary, mitochondria and mitochondria-associated molecules and pathways may be attractive targets to overcome TKI resistance in CML.

## 1. Introduction

Chronic myeloid leukemia (CML) results from the reciprocal translocation between chromosomes 9 and 22, t(9;22)(q34;q11), in a fraction of hematopoietic stem cells (HSC), producing a fusion chromosome, the Philadelphia (Ph) chromosome [1]. The Ph chromosome contains the fusion *BCR/ABL* gene which consists of fragments of the *BCR* and *ABL* genes, from the chromosomes 22 and 9, respectively. The product of this gene, the BCR/ABL protein, displays a constitutively high tyrosine kinase activity and confers some growth advantages to the Ph-positive clone [2]. This induces expansion of leukemic progenitor cells, which results in a clinically detectable CML in a rather slow chronic phase (CP), which in turn, if not treated, progresses to an accelerated (AP) and/or acute (blast, BP) phase. CML patients in the BP phase have bad prognosis with median survival time of several months. A population of CML cells consists of heterogeneous cell types at various maturation stages that are maintained by a small number of cells called CML stem cells, which are able to self-renew and proliferate. The introduction of imatinib mesylate (Imatinib, Gleevec, STI571, IM) was a milestone in CML therapy. IM belongs to tyrosine kinase inhibitors (TKIs) and induces complete cytogenetic response (CCR) in about 80% of CP patients. However, most patients with CCR have the *BCR/ABL* transcript, thus lacking complete molecular response (CMR) [3]. Moreover, patients with CMR face CML recurrence after stopping IM therapy [4]. This suggests: (i) presence of IM-sensitive patients with no detectable *BCR/ABL* transcript; (ii) presence of a small population of TKI-resistant Ph-positive stem cells; and (iii) lack of *BCR/ABL* transcript production. The latter, however, is resumed after withdrawal of IM.

**Figure 1:**
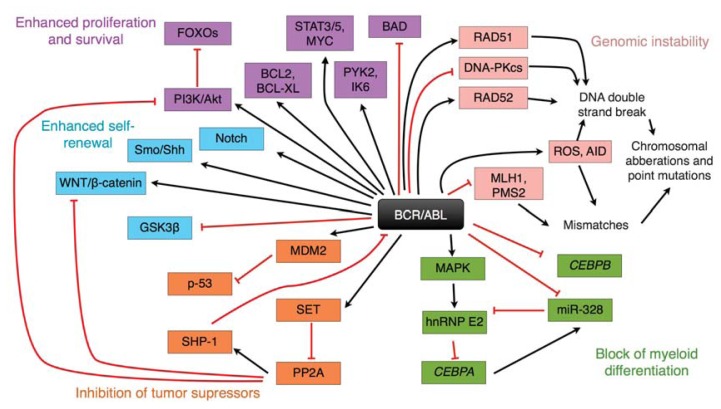
BCR/ABL plays an important role in cellular signaling involved in growth, proliferation, genomic stability, cancer transformation and survival. ROS—reactive oxygen species. Only some of the many signaling pathways, in which BCR/ABL is involved, are presented. (Adapted with permission from reference [6], copyright 2010 American Society for Clinical Investigation.)

Many signaling pathways cross at BCR/ABL in hematopoietic cells, including signal to inhibit apoptosis (Figure 1) [5]. As mitochondria may be involved in apoptotic processes, the stability of mitochondrial DNA (mtDNA) may negatively influence apoptotic signaling, possibly leading to resistance to proapoptotic signals associated with TKIs activity, as a result of DNA damage repairing deficiencies. Therefore, mitochondrial mutagenesis, including damage to and repair of mtDNA, may be important for TKI-based CML therapy.

## 2. Imatinib Resistance

There are several forms of resistance to IM-based therapy in CML patients. One is related to inherent IM-resistance of leukemic stem cells (LSC). Such cells form a residual population of cancer cells that can survive therapy. If IM therapy is then stopped, they will rebuild the population of leukemic cells, thus causing disease relapse [7]. The exact mechanism of this resistance is not fully understood, however dormancy of LSC, as well as their independence of BCR/ABL activity, may provide possible explanations [7,8]. Some patients exhibit primary resistance that renders them unsusceptible to IM therapy. One possible mechanism of such primary resistance is based on point mutations in the *BCR/ABL* gene that prevents IM binding [9–11]. Thus, cells with such mutations are resistant to proapoptotic action of IM [10]. Over fifty of such mutations were identified to date, each affecting IM binding to various extents. Two such mutations are Tyr253His and Thr315Ile [12]. Point mutations may alter BCR/ABL protein in the activation loop, phosphate binding P-loop, and the ATP-binding cleft [12]. However, further research led to the discovery of new generations of TKIs that can overcome some forms of resistance to IM resulting from point mutations. The second generation TKI nilotinib (formerly AMN107) is over thirty-fold more potent than IM and can suppress 32 of 33 mutant forms of BCR/ABL resistant to IM [13,14]. The other second generation TKI dasatinib (formerly BMS354825) and third generation bosutinib (formerly SKI-606) and ponatinib (formerly AP24534) were also found to overcome most of the BCR/ABL mutations related to IM resistance [15,16].

Some patients may develop resistance to IM in the course of therapy. In such cases, the mechanism of such development may consist of acquiring the relevant mutations in the *BCR/ABL* gene, its amplification, changes in signal transduction independent of BCR/ABL or changes in drug influx [17,18]. Altogether, lack of response or relapse to imatinib may be underlined by:

- mutations in the BCR/ABL1 kinase domain preventing the binding of imatinib
- clonal evolution
- pharmacokinetic variability
- amplification of the *BCR/ABL* gene
- overexpression of drug transporter genes
- overexpression of tyrosine kinases

Mutations in the BCR/ABL kinase domain have been detected in about half of the CML patients displaying imatinib resistance [19]. Such mutations affect BCR/ABL residues making direct contact with imatinib or stabilize/destabilize the active/inactive conformation of the kinase [17].

In attempts to fight IM resistance in CML cells, TKIs were combined with other substances promoting apoptosis and replaced by oxidative stress-inducers, including adaphostin and phenethyl isothiocyanate (PEITC) [20–22]. These substances exert cytotoxic effects by degradation of wild-type and mutated BCR/ABL, which is particularly important for CML cells harboring the T315I mutation. We and others showed that BCR/ABL could promote reactive oxygen species (ROS) generation and redox imbalance [23–25]. This may lead to oxidative stress in CML cells, resulting in their damage since BCR/ABL contains redox-sensitive cysteine residues that may be oxidized by ROS, causing changes in its structure and stability as a consequence. It was speculated, that the induction of a severe oxidative stress in CML cells by exogenous substances might potentiate general unfavorable conditions leading to BCR/ABL degradation [22]. This suggestion was confirmed in IM-resistant leukemia cell lines and T315I-positive CML cells isolated from CML patients [22].

## 3. Mitochondrial DNA Damage and Repair

Mitochondria are essential organelles serving as a source of metabolic energy in the form of ATP generated by oxidative phosphorylation (OXPHOS). Such organelles are also involved in a number of other key processes, such as signaling, cellular differentiation, cell death, as well as the control of the cell cycle and cell growth [26].

In aerobic cells, mitochondrial respiration is one of the major endogenous sources of ROS. In the mitochondrial electron transport chain (ETC), there is a sequential transfer of electrons, with cytochrome oxidase (COX) being the terminal acceptor which reduces molecular oxygen to water [27]. ROS are normal by-products of cell metabolism through mitochondrial respiration or several oxidases, such as nicotinamide adenine dinucleotide phosphate oxidase, xanthine oxidase, cyclooxygenase and lipoxygenase. ROS are required for maintaining normal cellular function, including signal transduction. It is estimated that up to 4%–5% of consumed mitochondrial oxygen is converted to superoxides and H_2_O_2_ [28]. ROS are usually eliminated by antioxidant enzymes, including superoxide dismutase, catalase, glutathione reductase and glutathione peroxidase. ROS, if not eliminated, can cause damage to DNA, lipids and proteins and have been implicated in a wide range of pathological conditions, including premature aging and cancer [29,30].

Mitochondrial DNA (mtDNA) is not associated with proteins in such a firm and organized fashion as nuclear DNA (nDNA), so it is particularly prone to oxidative damage compared with its nuclear counterpart, also due to its close location to endogenously generated ROS [31]. In addition, mtDNA does not contain introns and undergoes transcription at a high rate, resulting in a high probability of oxidative modification of DNA bases in the coding region. When mitochondria are damaged, the level of OXPHOS is decreased, but ROS production is usually increased [32]. It was shown that mitochondrial dysfunction could lead to altered gene expression, apoptosis and loss of cell viability [28,33,34]. ROS can cause a variety of DNA damage, including single and double strand breaks (SSBs and DSBs, respectively) and DNA base modifications. Therefore, efficient repair of ROS-induced DNA damage is important for preventing mutations and maintaining the stability of the mitochondrial genome [35].

**Figure 2:**
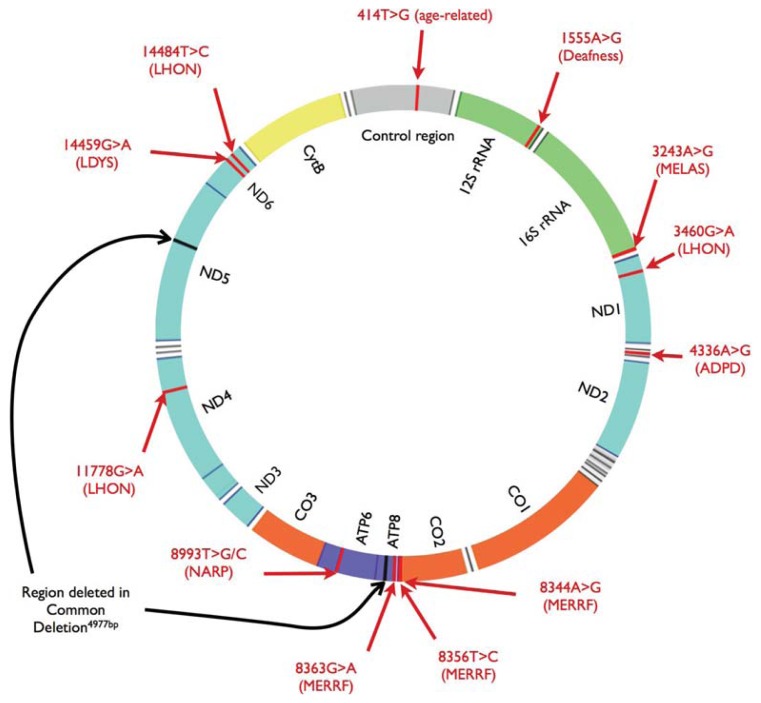
Organization of the human mitochondrial genome. Some of the most common mutations and related disorders are marked. rRNA—ribosomal RNA; ND1–6—NADH dehydrogenase subunits; CO1–3—Cytochrome oxidase subunits; ATP6 and ATP8—ATP synthase subunits; CytB—Cytochrome *b*; MELAS—Mitochondrial encephalomyopathy, lactic acidosis, and stroke-like episodes; LHON—Maternally inherited Leber’s hereditary optic neuropathy; ADPD—Alzheimer and Parkinson diseases; MERRF—Myoclonus epilepsy with ragged red fibers; NARP—Neurogenic muscle weakness, ataxia, and *retinitus pigmentosa*; LDYS—LHON associated with dystonia. Location of common deletion in mtDNA is also indicated (Adapted from reference [49]).

In general, mtDNA damage in the form of base substitutions, rearrangements, insertions and deletions may have serious phenotypic consequences, particularly because mtDNA encodes for 13 OXPHOS proteins, 22 transfer RNAs and two ribosomal RNAs (Figure 2). The most common disease causing point mutation in mtDNA is the transition m.3243A>G in the *MT-TL1* gene [mitochondrially encoded tRNA leucine 1 (UUA/G)], which is associated with maternally inherited diabetes and deafness [36,37] and MELAS (mitochondrial myopathy, encephalopathy, lactic acidosis, and stroke-like episodes) [38]. Another common transition, m.8344A>G in *MT-TK* (mitochondrially encoded tRNA lysine), causes myoclonic epilepsy associated with ragged-red fibers [39]. The m.6267A>G mutation that induces the Ala122Thr substitution in the mitochondrial-encoded cytochrome *c* oxidase I (MT-CO1) is associated with its reduced activity and tumors [40]. The m.8993T>G transversion in the *MT*-*ATP6* gene may be associated with prostate tumor [41] and progressive neurodegenerative disorder NARP (neurogenic muscle weakness, ataxia, and retinitis pigmentosa) [42]. The most common large deletions in mtDNA are deletions ranging from 8469 to 13,446 bp and from 8648 to 16,085 bp found in patients with myopathies [43]. Variations in mtDNA copy number may also be caused by mutations in the D-loop regulatory region or decreased activity of the mitochondrial polymerase γ [44]. It was shown that an increase in mtDNA copy number is associated with chronic lymphocytic leukemia (CLL), Burkett lymphoma, Epstein-Barr virus-transformed lymphoblastoid cell lines, non-Hodgkin’s lymphoma, and small lymphocytic lymphoma [45].

Due to the mutagenic nature of many ROS-induced lesions, there is a higher mutation rate in mtDNA than in nDNA and repair of these lesions may be crucial for the fate of mitochondria. No nucleotide excision repair (NER) pathway, which is essential for the stability of the nuclear genome, has been observed in mitochondria, although the presence of some NER-like mechanisms cannot be definitely excluded. Mismatch repair (MMR) and base excision repair (BER) have been reported to operate in mtDNA. Also, the activity of O^6^-methyl-guanine DNA methyl transferase (MGMT) was observed in mitochondria [46]. Homologous recombination repair (HRR) and nonhomologous end-joining (NHEJ) repair are critical for repairing DNA double-strand breaks (DSBs). Homologues of some proteins involved in these pathways in bacteria, yeasts and mammals have been identified in mitochondria and certain lesions, including cisplatin inter-strand crosslinks, which are repaired by recombinational pathways in the mammalian mitochondria [46]. MSH2 is the only protein involved in MMR that was found in mammalian mitochondria [47]. Mitochondria from rat liver MSH2-deficient cells displayed normal MMR activity, which suggests that mitochondrial MMR is underlined by a different mechanism than its nuclear counterpart [48].

The BER pathway is present in both the nucleus and mitochondria with similar mechanisms but different components [50]. The process of short-path BER is divided into four steps. A DNA lesion is recognized by a specific glycosylase, mostly by αOGG1 splice variant or mitochondrial UNG1, that cleaves the *N*-glycosidic bond, creating an abasic site (AP), which is processed by mitochondrial AP endonuclease (mtAPE) resulting in a single-strand break with a 3′-hydroxyl end and a 5′-deoxyribose-5-phosphate (5′-dRP) residue. This gap is filled by mitochondrial pol γ, which possesses a 3′–5′ exonuclease and a 5′dRP lyase activities. Remaining nick is sealed by mitochondrial DNA ligase III, produced by a splice variant of the *LIG3* gene, encoding both the nuclear and mitochondrial enzymes. In long-patch BER, pol γ forms 2–20 nucleotide-long fragments during repair synthesis, creating a flap structure processed by the flap endonuclease 1 (FEN-1). Since FEN-1 was detected in mitochondria, it is postulated that long-patch BER is present in these organelles [51].

The lack of NER and no solid evidence for the presence of DNA double strand repairing pathways suggest that damage to mtDNA may be less efficiently repaired than the same damage in nDNA [52].

## 4. Mitochondrial-Dependent Apoptosis

**Figure 3:**
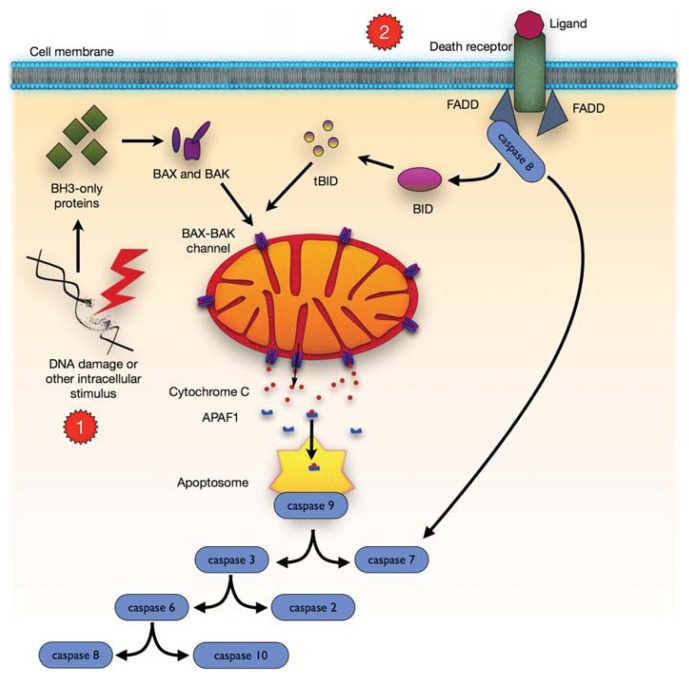
Comparison of the intrinsic and extrinsic apoptotic pathways. (1) *Intrinsic pathway*: Numerous internal stimuli, including DNA damage, activate BCL-2 homology 3 (BH3-only) proteins leading to BCL-2-associated X protein (BAX) and BCL-2 antagonist or killer (BAK) activation. This creates BAX-BAK channels in the mitochondrial outer membrane and causes its permeabilization which leads to the release of proapoptotic factors from the intermembranal space, including cytochrome *c*, which induces the apoptotic protease activating factor 1 (APAF1), thereby forming the apoptosome promoting the proteolytic maturation of activator caspase 9. Then, caspase 9 cleaves and activates other effector caspases—2, 3, 6–8, 10, eventually leading to apoptosis; (2) *Extrinsic pathway*: The first step is an association of death receptors with their cognate ligands, which leads to the recruitment of adaptor molecules, including FAS-associated death domain protein (FADD), and then caspase 8. Caspase 8 is able to directly cleave and activate caspase 3 and caspase 7 and can proteolytically activate BH3-only protein BH3-interacting domain death agonist (BID). Proteolytically activated BID (tBID) promotes mitochondrial membrane permeabilization through the activation of assembly of BAX-BAK channels and represents the main link between the extrinsic and intrinsic apoptotic pathways.

Apoptosis involves a sequence of events that decompose cells in a strictly regulated way (Figure 3). The extrinsic apoptotic pathway is mediated by death receptors that react with external death signals. Intrinsic (mitochondrial) pathway is mediated by the Bcl-2 family proteins [53].

DNA damage, ischaemia, and oxidative stress, are apoptotic signals that lead to cell death by the mitochondrial pathway. As a part of signaling in the intrinsic pathway, mitochondria release soluble proteins, including cytochrome *c*, from the intermembranal space to initiate caspase cascade in the cytosol [54,55]. The release of these proteins is a consequence of disturbed integrity of the mitochondrial outer membrane (OMM). This process is referred to as the mitochondrial outer membrane permeabilization (MOMP). In vertebrates, MOMP is under the control of proapoptotic Bcl-2 family members, including Bax, Bak and Bok/Mtd as well as BH3-only proteins, including Bid, Bim/Bod, Bad, Bmf, Bik/Nbk, Blk, Noxa, Puma [53,56].

## 5. Relationship between Chronic Myeloid Leukemia and Mitochondrial Mutagenesis

Prevention of proliferation of CML cells in the IM era was thought to require a continuous supply of the inhibitor. However, a long-term experiment with IM and other TKIs brought surprising results, indicating that a transient, but potent inhibition of BCR/ABL with IM, is sufficient to commit CML cells irreversibly to apoptosis [57]. Previously we showed that IM induced DNA damage in BCR/ABL-expressing cells, but did not do so in normal cells [58]. These results are especially interesting if they are linked with the recent point of view on the process of apoptosis, according to which, the process in a single cell is turned on after just once reaching a particular threshold, where there is a need to keep the threshold value continuously [56]. This reaching of the threshold occurs when antiapoptotic proteins of the Bcl-2 family, including Bcl-2, Bcl-XL or Mcl-1, are prevented from binding to the proapoptotic Bcl-2 family members Bax or Bak, which are then free to self-associate and form pores in the outer mitochondrial membrane [59]. This last step is the one that makes the irreversible commitment to cell death. Therefore, any perturbation in the functioning of mitochondria may result in change in the response of BCR/ABL-positive cells to IM. ROS are important regulators of apoptosis. At physiological levels they may function as redox messengers, but their increased levels may induce detrimental effects, including cell death [26]. This leads to a question of the possible role of mitochondrial ROS production in modulation of cancer development. There is some evidence of the direct role of mitochondria in promoting carcinogenesis. One of the well documented sequence of events consists of hypoxia that leads to a transient increase in ROS levels produced in mitochondria and the use of glycolysis as a major source of ATP [60,61]. Consequently, most cells go into apoptosis or senescence. However, a few cells may endure such increased levels of ROS. Such stabilization is possible through increased HIF-2alpha expression [62]. Thus, they are further exposed to mutagenic action of ROS and may become genetically unstable [60]. Leukemic cells with the active BCR/ABL kinase may activate pathways of increased glucose metabolism [24]. Moreover, IM action may induce elevated ROS levels in leukemic cells that also leads to genomic instability, including self-mutagenesis of *BCR/ABL,* which may lead to development of BCR/ABL variants resistant to IM [25].

ROS-induced mutations may affect the activity of genes present in the mitochondrial genome. The first intragenic subunit ND1 deletion (264 bp) was reported in renal cell carcinoma [63]. In another study, the common 4977 bp deletion in mtDNA was detected in 17 out of 32 (53%) primary gastric carcinomas [64]. There is clear evidence that the 13,997 G>A mutation in the *ND6* gene can control and promote metastasis [65]. Other reports suggest an association between the copy number of mitochondrial genome and the level of ROS production. It was hypothesized that increased copy number is an effect of a compensation mechanism that may replace damaged mitochondrial genomes with undamaged ones through replication [60,66]. Effective repair of oxidatively damaged mtDNA is essential, because mtDNA is located near the respiration chain and contains significantly higher levels of accumulated oxidative damage than its nuclear counterpart [67]. Moreover, it contains genes of some of the respiratory chain components. Thus, defective mtDNA that is translated into defective mitochondrial components, can further aggravate mitochondrial dysfunction by accelerating aberrant ROS production, inefficient ATP generation and ROS-induced self-mutagenesis. This can then result in oxidative DNA and protein damage and, in consequence, the start of a vicious cycle of events [60]. Therefore, cancer development may be associated with increasing levels of mtDNA damage accumulation [67,68]. However, there is a lack of research aimed at investigating such a relationship in CML. It was shown that chronic lymphocytic leukemia patients had a higher level of mtDNA damage after chemotherapy. In addition, those patients that were resistant to conventional chemotherapy showed even higher levels of mtDNA oxidative damage accumulation compared to those susceptible to therapy [69]. Some of those patients carried mutations in the cytochrome *c* oxidase II gene associated with increased levels of ROS production [69]. This further supports the vicious cycle hypothesis and its role in cancer development or resistance to therapy.

Similar results were obtained for patients with acute myeloid leukemia, in which a variation in genes encoding cytochrome *c* oxidase subunit I and II, as well as in the control region of mitochondrial genome, was correlated with inferior disease-free survival [70]. Moreover, there are reports on an association between increased mtDNA copy number and the risk of non-Hodgkin lymphoma [45]. In addition, some results indicated that leukemic cells could contain more dimeric forms of mtDNA than normal cells, and this difference decreased after treatment with anti-leukemic drugs [71].

Research on aging shows that accumulation of mutations in mtDNA is correlated with such a process, along with increased levels of apoptosis markers [72]. It suggests that mtDNA damage may promote susceptibility to pro-apoptotic signals sent by TKIs. This is further supported by the observation that cells lacking expression of some mitochondrial genes exhibit a significantly higher tendency to undergo apoptosis *in vivo* [73]. Results of the studies on the effects of homo- and heteroplasmic mutations in the *ND5* gene on cancer development present a complex interaction. Though homoplasmic mutations increase the probability of apoptosis, heteroplasmic mutations may have an adverse effect and promote tumor growth [74]. There is also an important observation of induced chemoresistance to bile acids in hepatocytes. It was demonstrated that hepatocytes depleted of mtDNA constantly activated the pro-survival Akt/mTOR pathway, making them resistant to apoptosis after exposure to bile acids [75]. Activation of Akt/mTOR pathway is also an important feature of cells with BCR/ABL kinase activity [24]. This suggests possible involvement of mtDNA damage in survival of CML after IM treatment. In fact, such a mechanism has already been detected in some other cancers [61].

## 6. Mitochondria and Resistance to Tyrosine Kinase Inhibitors

It was shown that Rac2 GTPase disturbed mitochondrial membrane potential and electron flow through the complex III of the mitochondrial respiratory chain in leukemic stem cells of CML CO and primitive leukemic progenitor cells [76]. These disturbances resulted in an enhanced ROS production, supporting genomic instability and, in consequence, accumulation of mutations. Some of these mutations might contribute to TKI resistance. ROS-induced genomic instability was reduced by the expression of mitochondria-targeted catalase and the addition of a mitochondria-targeted peptide aptamer neutralizing ROS. An enhanced ROS production might lead to depolarization of mitochondrial membrane, resulting in diminished ATP production and release of cytochrome *c*. Although the cytochrome has proapoptotic properties, its activity may be hampered by the antiapoptotic action of BCR/ABL [77].

It was shown that BMS-214662, a farnesyl transferase inhibitor, induced apoptosis via the intrinsic pathway, associated with a loss of mitochondrial membrane potential, condensation and swelling of the mitochondria in the perinuclear region, mitochondrial outer membrane permeabilization, release of cytochrome *c* from the mitochondria, and ROS production [78]. Similar effects were observed in other research [79,80].

The “mitochondrial connection” in IM resistance is also suggested by the results showing an enhanced level of glycolysis in IM resistant cells [81]. In general, *BCR/ABL* expression may stimulate glucose uptake by hematopoietic cells, which is underlined by translocation of transporters of glucose assisted by the PI3 kinase [82,83]. IM apparently changes the metabolic status of BCR/ABL-positive cells and it may be involved in controlling the glucose flux by affecting dissemination of the glucose transporter GLUT1 [84–86]. The tricarboxylic acid cycle, a key element of aerobic respiration, was reported to be changed by clinically relevant concentrations of IM, which led to activation of certain mitochondrial functions [87]. However, TKI-resistant cells had a high level of glycolysis both in the presence and absence of IM, suggesting that elevated glucose metabolism may be involved in TKI resistance in CML cells [81,88]. This high level of glucose metabolism in BCR/ABL-positive cells may lead to overproduction of ROS, resulting in such a mitochondrial pathway of TKI resistance [24].

In general, a growing body of evidence suggests that BCR/ABL-positive cells may use glycolysis to supply ATP needed to proliferate [87,88]. It was also shown that the expression of some genes involved in glycolysis, including pyruvate dehydrogenase kinase and cytochrome *c* oxidase subunits, were increased in IM-resistant CML cells [88]. These genes are mostly regulated by HIF-1α, which may be linked with IM resistance [88,89]. The involvement of HIF-1α and the impairment of the mitochondrial respiratory system, as suggested by the Warburg hypothesis, in IM resistance, may confirm the importance of mitochondria for this effect [90]. It was hypothesized that a high level of glucose production may be involved in IM resistance through the inhibition of p53 inactivation [91]. In summary, it may be hypothesized that inhibiting glucose metabolism by targeting some mitochondria-associated molecules/processes, may increase the effectiveness of TKI-based CML therapy as well as by overcoming TKI resistance.

## 7. Conclusions

**Figure 4:**
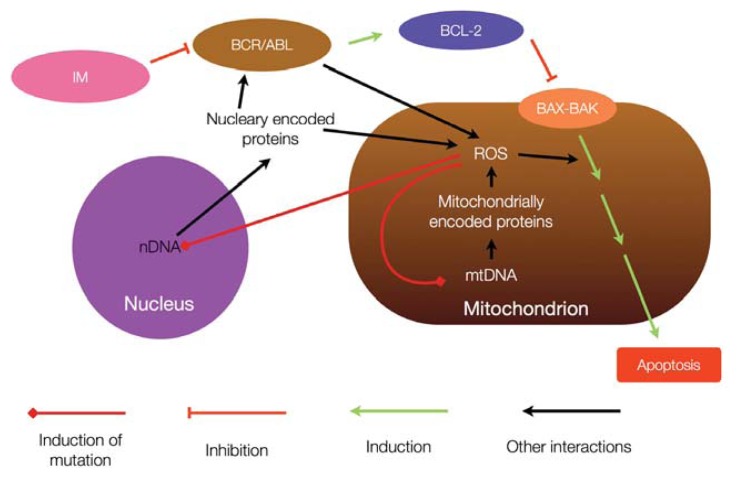
Interaction between mtDNA and BCR/ABL and its role in imatinib mesylate (IM)-resistance. BCR/ABL can promote mtDNA mutation through induction of ROS production. This may further alter mitochondrial metabolism, not only affecting susceptibility to apoptosis, but also inducing nuclear DNA mutagenesis. Such mutagenesis may subsequently lead to novel forms of BCR/ABL, resistant to IM action.

Mutagenesis of mtDNA and altered mitochondrial metabolism may be associated with several cancers and this association may be underlined by altered susceptibility to apoptotic signals. This altered susceptibility may also contribute to cancer cell resistance to apoptosis-induced therapy, which may be the case in the resistance of CML cells to TKIs (Figure 4).
